# Sleep and sleep-related breathing disorders in patients with spinal muscular atrophy: a changing perspective from novel treatments?

**DOI:** 10.3389/fneur.2024.1299205

**Published:** 2024-06-04

**Authors:** Elena Abati, Eleonora Mauri, Martina Rimoldi, Barbara Madini, Francesca Patria, Giacomo Pietro Comi, Stefania Corti

**Affiliations:** ^1^Neurology Unit, Department of Neuroscience and Mental Health, Foundation IRCCS Ca’ Granda Ospedale Maggiore Policlinico, Dino Ferrari Centre, Milan, Italy; ^2^Department of Pathophysiology and Transplantation (DEPT), University of Milan, Milan, Italy; ^3^Neurophysiopathology Unit, Department of Neuroscience and Mental Health, Foundation IRCCS Cà Granda Ospedale Maggiore Policlinico, Milan, Italy; ^4^Pediatric Pneumonology, Foundation IRCCS Cà Granda Ospedale Maggiore Policlinico, Milan, Italy; ^5^Neuromuscular Disease Unit, Department of Neurosciences and Mental Health, Foundation IRCCS Cà Granda Ospedale Maggiore Policlinico, Milan, Italy

**Keywords:** spinal muscular atrophy, sleep, respiratory disorders, sleep-related breathing disorders, non-invasive ventilation

## Abstract

Spinal Muscular Atrophy (SMA) is an inherited neuromuscular disorder characterized by progressive muscle weakness and atrophy, resulting from the degeneration of motor neurons in the spinal cord. A critical aspect of SMA is its impact on respiratory function. As the disease progresses, respiratory muscles, in particular intercostal muscles, become increasingly affected, leading to breathing difficulties and respiratory failure. Without intervention, many children with SMA type 1 die from respiratory failure before their second year of life. While assisted ventilation has improved survival, it often results in ventilator dependence. The development of new SMN-augmenting therapies has renewed optimism, but their long-term impact on respiratory function is uncertain, and non-invasive respiratory support remains an important part of SMA management. Despite the importance of respiratory support in SMA, knowledge regarding sleep disorders in this population is limited. This review aims to synthesize existing literature on sleep and sleep-related breathing disorders in patients with SMA, with a focus on SMA type 1. We summarize evidence of sleep-disordered breathing and respiratory failure in SMA, as well as outcomes and survival benefits associated with non-invasive or invasive ventilation with or without pharmacological therapies. We also discuss current knowledge regarding the effects of novel disease-modifying therapies for SMA on respiratory function and sleep. In conclusion, optimal care for children with SMA requires a multidisciplinary approach that includes neurology and respiratory specialists. This review highlights the importance of monitoring sleep and respiratory function in SMA, as well as the potential benefits and challenges associated with assisted ventilation combined with new therapies.

## 1 Introduction

Spinal Muscular Atrophy (SMA) is a rare autosomal recessive genetic disorder that affects the motor neurons in the spinal cord and brainstem (1–4). The *SMN1* gene is responsible for the production of the SMN (survival motor neuron) protein, which plays a crucial role in motoneuronal function (5). Induce aberrant SMN1 splicingresulting in progressive degeneration and loss of motor neurons, with subsequent muscle weakness and respiratory impairment. The *SMN2* is a paralogous gene of SMN1 lacking exon 7, thus the corresponding mRNA is translated into an unstable and non-functional protein, that cannot compensate for the *SMN1 loss* (5–9).

*SMN2* can be present in a variable number of copies, which in turn affect the residual activity of the SMN protein. SMN production levels and *SMN2* copy number can predict the clinical course of SMA, whose severity can thereby widely vary. SMA clinical classification is based on age of onset and maximum motor function achieved (1, 4, 10–14). SMA type 0 is a very severe form with reduction of fetal movements. SMA type 1 patients manifest symptoms before age 6 months and never acquire the sitting position. SMA type 2 has onset between 6 and 18 months with hypotonia, achieve motor milestones up to 6 to 8 months of age, they are able to sit if placed in position but not to walk. In SMA type 3 symptoms onset is after age 18 months and acquire independence in ambulation and may have a normal life expectancy (4, 11). SMA type 4 is an adult form with mild symptoms (14). The gold standard of SMA genetic testing is a quantitative analysis of both *SMN1* and *SMN2* genes (15). *SMN1* testing is relevant for the identification of heterozygous deletions or mutations, while *SMN2* copy number evaluation is important for prognosis and therapeutic approaches. Currently, three authorized treatments which are able to increase SMN protein levels are available for SMA patients, namely nusinersen (Spinraza), onasemnogene abeparvovec (Zolgensma), and risdiplam (Evrysdi). Nusinersen is an antisense oligonucleotide targeting *SMN2* gene that has been approved by the FDA and by the EMA for the treatment of all types of SMA. Onasemnogene abeparvovec is a gene therapy approved for the treatment of pediatric patients less than 2 years of age with bi-allelic mutations in the *SMN1* gene (FDA) with either a clinical diagnosis and up to three copies of the *SMN2* gene (EMA). Risdiplam is a small molecule that was recently approved for the treatment of pediatric and adult SMA patients by the FDA; EMA indications are restricted to patients 2 months of age and older, with a clinical diagnosis of SMA and one to four *SMN2* copies. These therapies have led to a profound transformation in the paradigm of SMA prognosis and progression. Given the intricate nature of the condition, a multidisciplinary methodology remains the key to enhancing both the quality of life and the lifespan of SMA patients (16–18).

Breathing disorders are a prevalent yet often undetected issue causing sleep disruptions and daytime symptoms in children with SMA (19). Several studies have investigated the consequences of muscle weakness s in SMA, highlighting how they may lead to nocturnal hypoventilation, respiratory muscle weakness and frequent pulmonary infections due to insufficient clearance of airway secretions, and sleep fragmentation, with overall negative impact on sleep quality (20). Without intervention, many children with SMA type 1 die from respiratory failure before their second year of life. While assisted ventilation has improved survival, it often results in ventilator dependence. The development of new SMN-augmenting therapies has renewed optimism, but their long-term impact on respiratory function is uncertain, and non-invasive respiratory support remains an important part of SMA management. Helping SMA individuals to achieve their best possible quality of life is an essential part of health care (18). Tailored management approaches, encompassing respiratory assistance, positioning assistance, and lifestyle adaptations, play a pivotal role in enhancing sleep quality and the holistic wellness of individuals with SMA.

This review aims to synthesize existing literature on sleep and sleep-related breathing disorders in patients with SMA. We summarize evidence of sleep-disordered breathing and respiratory failure in SMA, as well as outcomes and survival benefits associated with non-invasive or invasive ventilation with or without new pharmacological therapies.

## 2 Respiratory function and sleep in SMA

### 2.1 Breathing disorders in SMA

The drive to breathe and the functioning of the respiratory muscles, known as the “respiratory pump,” efficiently fulfill the demands for ventilation during periods of rest, physical exertion, and sleep. Conversely, patients affected by neuromuscular disorders face the potential of respiratory failure due to an imbalance between insufficient muscle strength and, often, an increased respiratory load. In SMA, muscle weakness and atrophy involve mainly intercostal respiratory muscles, while the diaphragm is relatively spared (21, 22). Inspiration movement in SMA patients is driven by the diaphragm, causing a negative intrathoracic pressure not balanced by weak upper rib cage muscles, leading to the abnormal conformation of the bell-shaped chest while paravertebral muscle weaknesscauses kyphoscoliosis (22). As a consequence of weakness and abnormal application of the forces, Tidal volume (TV) is decreased, so breathing frequency increases to maintain metabolic supply (21–29).

Particularly, SMA type 1 children present normal minute ventilation (VE) with an increase of Rapid and Shallow Breathing index (RSBi) and respiratory rate (RR), leading to a reduction in Tidal volume. In these patients’ category a normal ventilation is obtained in supine position by rapid and shallow breathing with paradoxical thoraco-abdominal motion. On the other hand, SMA type 2 children in seated position presented normal VE, higher RR and reduced TV resulting in rapid and shallow breathing, whereas in supine position they breathe like healthy peers (30).

The muscular dysfunction hampers the ability to generate sufficient negative intrathoracic pressure during inspiration and to effectively clear secretions from the airways. Consequently, individuals with SMA experience a decline in lung compliance and vital capacity, leading to a decreased ability to exchange gasses adequately and increasing the susceptibility to respiratory infections and respiratory failure (21, 22, 24, 26, 28, 29). With the progression of the disease, alveolar ventilation reduces and metabolic dysfunction as hypoxemia and hypercapnia manifests.

The severity of these complications varies across SMA types, with SMA type 1 typically exhibiting the most severe respiratory impairment, while SMA type 3 and type 4 may show milder but still significant respiratory challenges (20–23, 25, 27, 31).

The intricate interplay between motor neuron degeneration and respiratory muscle dysfunction underscores the necessity for vigilant monitoring and timely interventions to preserve pulmonary function and enhance the overall quality of life for SMA patients (29, 32).

### 2.2 Sleep disordered breathing in SMA

During sleep, intercostal and upper airway muscle activity decreases, particularly during REM-related hypotonia, functional residual capacity reduces and airflow resistance increases due to the supine position, thus ventilation-perfusion mismatch increases (33). In the REM phase, alveolar ventilation decreases. A study by Chacko and colleagues demonstrated an abnormality of sleep macrostructure and microstructure in SMA type 1 and 2 patients (34). Most sleep disordered breathing (SDB) occurs in SMA type 1 and type 2 patients, however this impairment should be considered in all children with SMA, including type 3 (34). Respiratory events are more frequent in REM sleep than in NREM for all types of SMA (34), possibly due to atonia of intercostal muscles during the REM phase (34). The hypothesized mechanism of central events is indeed the dysfunction of the respiratory muscle pump, secondary to early intercostal muscle weakness and progression to diaphragm weakness in advanced disease (34). No child exhibited obstructive sleep apnea alone, and this is consistent with the finding by Mellies and colleagues, which found that, although hypotonia of the pharyngeal muscles can lead to upper airway obstruction symptoms during REM sleep, this occurrence is infrequent among children diagnosed with SMA type 1 (19).

Moreover, in sleep, chemoreceptor sensitivity and central drive are less sensitive. At arterial gas analysis, the reduction of SpO2 and the increase of PaCO2 are common findings. For all these reasons, hypoventilation in SMA manifests earlier in the nighttime before the development of diurnal respiratory failure (33, 35, 36). Due to a physiological lower functional residual capacity, infants are more susceptible to oxygen desaturation (37). Moreover, the apnea/hypopnea index (AHI) is increased in SMA patients (24).

Since the respiratory effort is maximal in basal conditions, during respiratory tract infection SMA children are not able to increase the effort to sustain oxygen supply and respiratory failure occurs, requiring respiratory support. Furthermore, compromised clearance of secretions could also contribute to aberrant gas exchange (38).

Moreover, obstructive sleep apnea syndrome (OSAS) can manifest in SMA and lead to cardiovascular consequences in morbidity and mortality (39). SDB and OSAS typically serve as the initial indication of advancing respiratory dysfunction in individuals with SMA (40).

### 2.3 Sleep in SMA

In patients with SMA, sleep alterations concern both the cardiorespiratory system and the sleep structure stage architecture. Indeed, very few studies investigated sleep patterns in patients with SMA. Scattered reports of a few patients revealed a decrease or absence of REM sleep together with an increase of stage 1 sleep in SMA patients (41). Furthermore, sleep latency was found to be significantly prolonged in SMA type 1 patients, but no data are available regarding sleep latency in SMA type 2 and 3 (42). Regarding NREM sleep, N1 and N2 were increased in SMA type 1, and stages N1was increased in SMA type 2 (24, 41, 42), while slow-wave sleep (sleep stage N3) were decreased (42). However, stage N2 sleep proportion is found to be lower in SMA type 1 compared to types 2–3, and stages N1-3 increased parallelly with age (34).

REM sleep proportion was decreased in SMA patients (42), however it increased with decreasing age (34), thus its proportion was significantly greater in SMA type 1 compared to type 2, but not to type 3. Total sleep time was decreased in SMA type 2 patients and the sleep fragmentation was increased due to respiratory-related arousals (24).

Interestingly, sleep microstructure was described in SMA type 1 analyzing Cyclic Alternating Pattern (CAP) of non-REM sleep. Few sleep microarousals in NREM sleep despite a SDB were present (43). These findings, although the alterations described were less severe than those observed in SMA type 1, were confirmed also in children with SMA type 2 (44) and interpreted as a central nervous system failure to arouse (43, 44).

Impaired sleep architecture was improved by non-invasive ventilation, which allowed deeper sleep and prolonged REM sleep, reducing arousals at electroencephalogram (EEG) (19).

## 3 Monitoring sleep and breathing in SMA

**Figure 1:**
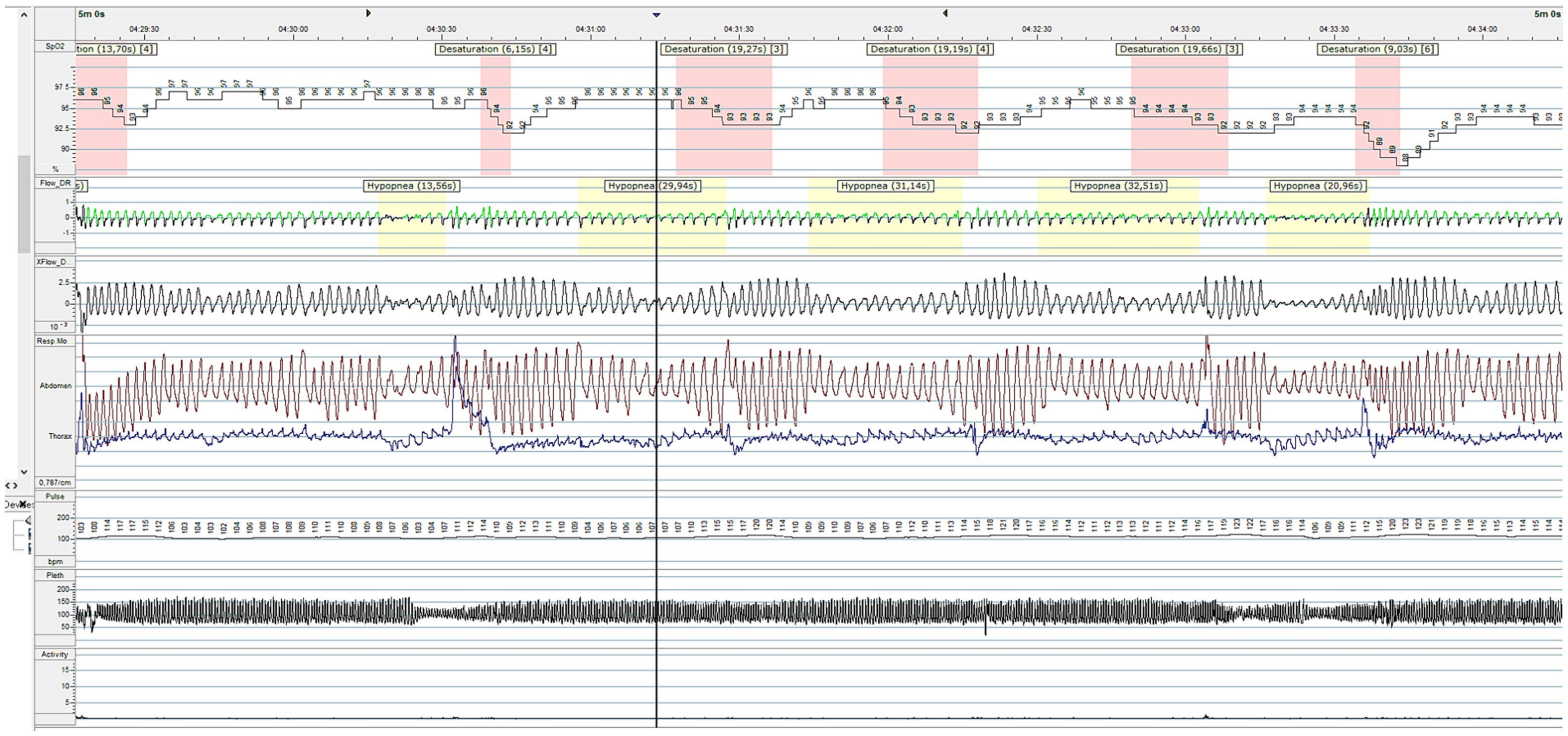
Respiratory polygraphy of an SMA patient showing a cluster of hypopneic events.

**Figure 2:**
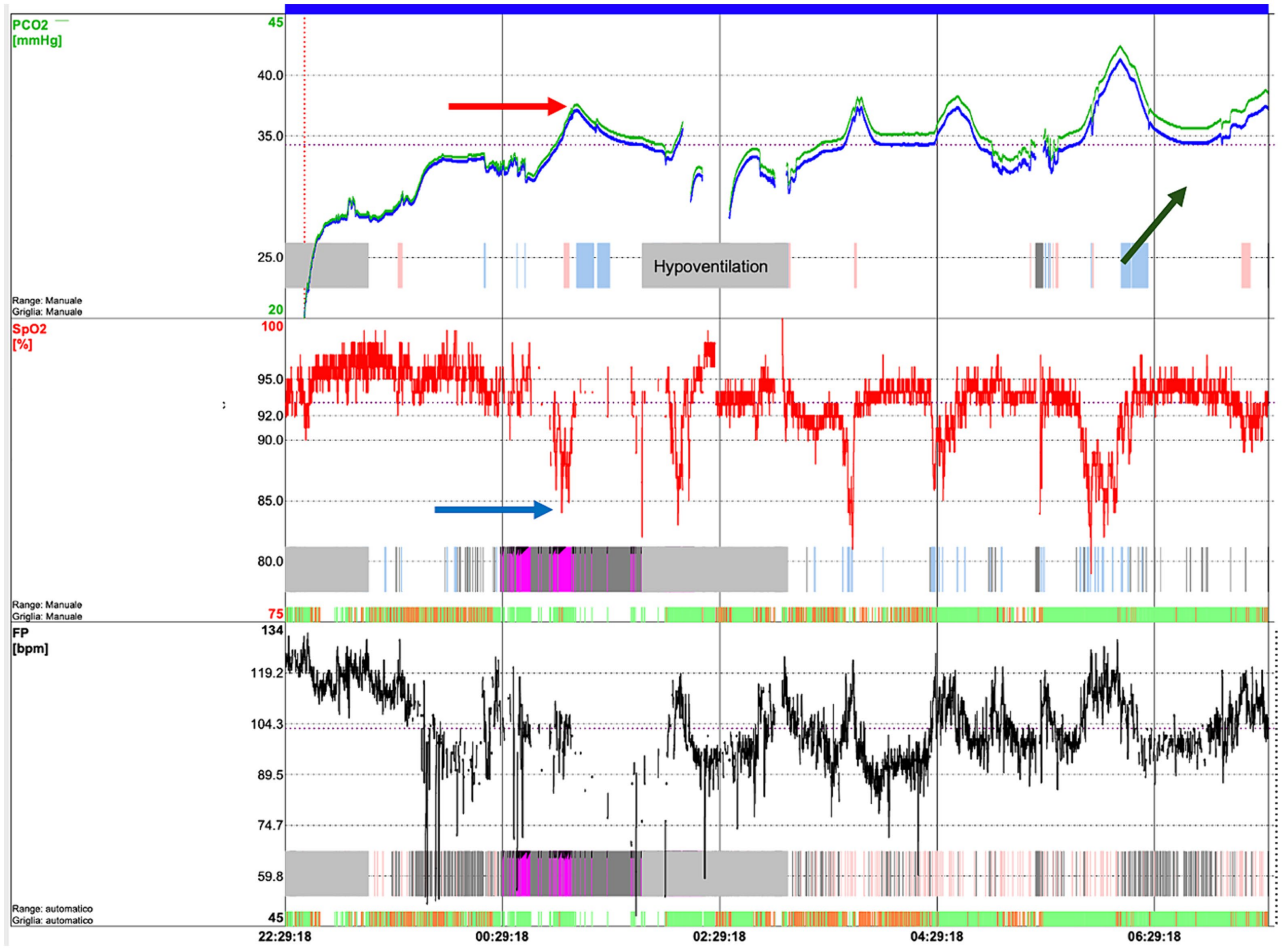
Transcutaneous capnography of an SMA patient showing hypoventilation episodes, in particular, desaturations (blue arrow) which correspond to an increase in CO2 (red arrow). In addition, an increasing trend in CO2 during the night can be observed (green arrow), associated with transition into REM sleep and muscle exhaustion.

To diagnose SDB, the quality of sleep and the need for non-invasive ventilation (NIV) are evaluated through cardiorespiratory polygraphy (CRSS) and overnight sleep polysomnography (PSG), according to BTS guidelines (45). CRSS consist of the simultaneous measurement of oxygen saturation, airflow, snoring, cardiac frequency, thoracic effort and body position through superficial electrodes; it has to be considered for diagnosing SDB in children, both those with and without comorbidity other than the respiratory issue. The addition of CO2 monitoring to pulse oximetry should be considered for children with comorbidities and suspected SDB where hypoventilation occurs, such as patients with neuromuscular disease (45). On the other hand, PSG is considered the gold standard for monitoring respiratory function (45) and diagnosing SBD such as OSA, and it serves as a valuable tool for assessing nocturnal seizures, narcolepsy, periodic limb movement disorder, and rapid eye movement sleep behavior disorder (46). It offers a comprehensive evaluation, detailing sleep architecture and arousals related to various events (24). It is a non-volitional and moderately invasive method since multiple polygraphic electrode channels are secured on the baby. The synchronous registration of the cerebral activity by adding electroencephalogram (EEG) channels can clarify differential diagnosis in central hypopnea (46). EEG refers to 10–20 system including at least six channels: Fp1, Fp2, C3, C4, O1, and O2; left and right electrooculogram (EOG); chin electromyogram (EMG); electrocardiogram (ECG); oronasal thermistor, snoring, nasal pressure transducer, thoracic and abdominal respiratory effort (inductance plethysmography); pulse oximetry-derived arterial hemoglobin oxygen saturation and pulse waveform, and transcutaneous partial pressure of carbon dioxide (24) (Figure 1). Indeed, it is time consuming and has to be performed in sleep study laboratories, which are not extensively distributed on the territory. While often conducted in a supervised, inpatient setting, PSG can also be effectively performed at home under proper supervision (45). PSG is non-volitional and moderately invasive since 16 polygraphic electrode channels are placed on the baby to simultaneously record heart rate, chest and abdomen movements, peripheral blood oxygen saturation (pulse oximetry) and nasal airflow. The AASM (American Academy of Sleep Medicine) has drafted guidelines for scoring and interpretation of the PSG. The scoring of respiratory events requires a certain degree of expertise, because of the peculiar sign of global inspiratory muscle weakness, different from the typical apneic or hypopneic events seen in patients affected by obstructive respiratory syndrome (OSA) (47, 48). These are represented by a decrease in thoracic and abdominal movements, a decrease in airflow, associated or not to the decrease in pulse oximetry (SpO2) and/or an increase in transcutaneous carbon dioxide (PtCO2) (47). To detect hypoventilation it is crucial to add ptCO2 measurement (Figure 2). However, the diagnosis of hypoventilation in literature is controversial: for the American Academy of Sleep Medicine, in patients affected by OSA, hypoventilation is a percentage of sleep time with a ptCO2 > 50 mmHg > 25% (48). Some authors believe that in patients with neuromuscular disease, considering such a large time interval could lead to excessive delay in starting NIV, so they suggest using for the diagnosis of hypoventilation a percentage of sleep time with ptCO2 > 50 mmHg > 2% (34, 49–51). In SMA patients, respiratory muscle weakness can manifest particularly in REM sleep, with periods of hypoventilation or paradoxical breathing (47).

In the study of Chacko et al. significant differences were described in the PSG respiratory parameters between types 1 and type 2 or type 1 and 3 SMA, particularly regarding Apnea-Hypopnea Index (AHI) and the Total Obstructive Index both more frequent in SMA type 1 versus type 2 and 3; no significant differences emerged between SMA type 2 and 3 (34).

## 4 Non-invasive ventilation in SMA

Effective management of respiratory complications in SMA involves a multi-faceted approach. Standard of care includes respiratory physiotherapy for airway clearance and, for most patients, nocturnal ventilatory support using noninvasive ventilation (NIV) (16). In the face of progressive respiratory deterioration, ventilatory support becomes a crucial intervention to ensure adequate breathing and oxygenation. NIV and invasive ventilation are the two main strategies employed to provide respiratory assistance to SMA patients. NIV pertains to the administration of positive pressure ventilation through a non-invasive interface, such as a nasal mask (38). In SMA patients, the primary objective of NIV is to assist the compromised respiratory muscles. This assistance is achieved by delivering a positive inspiratory pressure that can either augment the patient’s inherent breathing effort or substitute it in case of its absence (38). NIV, delivered through devices like bilevel positive airway pressure (BiPAP) is often the initial choice to support respiratory function. This method assists in maintaining lung volumes, preventing atelectasis, and alleviating hypoventilation during sleep (38, 52). Furthermore, NIV has an impact on prolonging survival and averting tracheostomy in SMA patients. However, as the disease progresses and the need for ventilatory support intensifies, invasive ventilation, such as tracheostomy, might become necessary. There is currently no clear validated indication on when to initiate NIV in patients with SMA. For most neuromuscular diseases, the decision to start NIV is based on nocturnal gas exchange recordings and the presence of nocturnal hypoventilation (BTS guidelines). NIV can be initiated in a chronic setting, after a sleep study showing nocturnal hypoventilation or in an acute-subacute setting, during an hospitalization for acute respiratory failure. The former setting is preferred as it may be less stressful for the patient and family. The goal should be to initiate NIV before hypercapnia and chronic daytime respiratory failure occur (33). Given that SMA type 1 patients frequently manifest evident indications of respiratory distress and heightened breathing effort even when gas exchange parameters remain within normal limits, certain experts advocate initiating NIV before the onset of respiratory failure, to alleviate dyspnea and prepare the patient for potential acute deterioration that might arise during intercurrent infections or instances of pulmonary aspiration, particularly in the presence of bulbar dysfunction (16, 32, 53). NIV can also be used as a preventive measure against the emergence of chest deformities (16, 32, 54–56).

The successful implementation of NIV among infants hinges on meticulous consideration of age-related and disease-specific factors. The appropriateness of mask fit stands as a critical determinant for the effectiveness of NIV. For children diagnosed with SMA type 1, characterized by bulbar dysfunction and the risk of aspirating oral secretions or gastric contents, nasal masks are the preferred interface (25). Equally pivotal is the selection of a suitable home ventilator device. The chosen ventilator should be capable of safely delivering low tidal volumes and incorporating an exceedingly sensitive flow trigger that enables synchronization with the patient’s breathing rhythm. A humidifier is usually placed between the ventilator and the interface to heat the air that is delivered to the patient. The preferred mode of ventilation is BiPAP, a pressure-mode ventilation, which deliver two levels of pressure, a higher pressure during inhalation to assist with breathing in and a lower pressure during exhalation to maintain airway patency without overexerting the respiratory muscles – inspiratory positive airway pressure (IPAP).

For non-sitters, typically SMA type 1 patients, NIV is introduced not just reactively when symptoms of respiratory failure become evident, but rather proactively (16). This shift toward early intervention aims to prevent or minimize respiratory complications and chest wall deformities that often accompany the progression of the disease. Regarding the specific settings of NIV, the initial focus is on ensuring comfort and efficacy. No specific guideline related to the settings of NIV in SMA has been issued so far. Settings should be individually tailored, taking into account the patient’s age, disease severity, and response to the therapy (16).

Experimental and clinical comparison to ensure a correct thoracic expansion recommend that a medium tidal volume of 8–10 mL/kg every respiratory act should be achieved (57). Sometimes, if the correction of hypoventilation is not achievable with the classic mode, it becomes necessary to use hybrid modes of ventilation, such as average volume-assured pressure support (58). Given the potential limitations posed by weakened children who might struggle to initiate inspiration or cycle into expiration even in the most sensitive settings, it is advised to incorporate a backup rate and fixed inspiratory time (25). Currently, many ventilators present the option of setting a minimum and maximum inspiratory time to minimize the possibility of patient-ventilator’s asynchrony (59).

In cases of neuromuscular disorders, the backup rate should be set in close proximity to the spontaneous breathing rate, typically around 2–3 breaths below, and generally not exceeding 30–35 breaths per minute (33, 60, 61). Following the establishment of effective ventilation, many children who previously exhibited tachypnea tend to synchronize with the backup rate, especially during sleep, in the absence of inspiration triggers (62).

In sitters, which include children with SMA types 2 and 3, NIV is also employed predominantly as a proactive measure (16). The settings and types of NIV used are similar to those in non-sitters, with adjustments made based on individual patient needs, tolerance, and the progression of their condition. The initiation of NIV in this group is often based on clinical assessments and results from spirometry tests and sleep studies. These assessments help determine the onset of nocturnal hypoventilation – a common initial sign of respiratory failure in SMA.

Precise adjustment of pressures and systematic evaluation of ventilator configurations are optimally achieved through polysomnography or respiratory polygraphy. These procedures not only give valuable insights into gas exchange and thoraco-abdominal asynchrony but also identify and enhance synchrony issues between the child and the ventilator (56, 63). In cases where these advanced tools are unavailable, the analysis of built-in software associated with oxygen and ptCO2 monitoring can be used to evaluate NIV. If the correction of hypoventilation remains uncertain, despite the use of those data, a complete sleep study is indicated (64).

When starting NIV, meticulous adaptation to the mask is pivotal to ensure subsequent tolerance. Ideally, the setup for nocturnal NIV should be done within a hospital environment under the guidance of an experienced team, accompanied by comprehensive training for the caregivers. Commencing with conservative pressure settings is advisable, which allow for subsequent titration based on either clinical benchmarks such as thoraco-abdominal asynchrony and augmented respiratory work or polysomnographic indicators like sleep-disordered breathing and gas exchange equilibrium. Some experts advocate the utilization of an abdominal belt to counteract thoracoabdominal asynchrony, ameliorate ventilation of upper lobes, and thwart abdominal distension; however, this recommendation is based solely on clinical observation (65, 66). Additionally, the choice of interface, which refers to the mask or other device used to deliver NIV, is of paramount importance. A proper fit is essential for both comfort and effectiveness. Often, two different interfaces with varying contact points on the face are recommended to prevent pressure sores and enhance tolerance.

The decision to initiate NIV in SMA patients is a multifaceted one. Indications for starting NIV include symptoms of respiratory distress, evidence of nocturnal hypoventilation, and declining pulmonary function tests. However, the timing of initiation and the aggressiveness of the intervention are influenced by factors such as the progression of SMA, the child’s overall health status, and the family’s goals for care.

## 5 Benefits and challenges associated with assisted ventilation

NIV has now gained widespread recognition as the primary approach for treating respiratory failure in children with SMA type 1 and its merits over tracheostomy and invasive ventilation have been acknowledged. NIV presents easier management for patients with stable respiratory conditions, alongside reduced technological demands. Additionally, the stark contrast is evident when considering the loss of independent breathing capacity observed in small and feeble children after tracheostomy, while their counterparts relying on NIV often retain a degree of unassisted breathing ability (67). Some authors suggest that even children with substantial reliance on ventilation—needing over 16 h of respiratory support daily—may be capable of certain periods of unsupported breathing, depending on their level of dependence (38). This underscores how NIV simplifies daily care and allows for ventilator independence. Notably, children with SMA type 1 and tracheostomy invariably lose vocalization abilities, whereas those on non-invasive support may retain speech abilities (68). Observational data indicate that long-term survivors utilizing NIV might require fewer hospitalizations over time compared to children on invasive ventilation (67). These cumulative factors contribute to an improved quality of life, although comprehensive systematic evidence remains pending publication.

Several studies have attempted to evaluate the potential advantages of nocturnal noninvasive ventilation concerning respiratory patterns during sleep, sleep quality, and daytime issues in pediatric patients with SMA. Available studies encompassed patients diagnosed with SMA type 1 subjected to non-invasive or tracheostomy-based ventilation. The provision of tracheostomy or noninvasive respiratory support emerged as pivotal, enabling SMA type 1 children to survive beyond the age of 2 (39). Notably, NIV emerged as a crucial factor associated with increasing survival in patients with SMA type 1, diminished hospitalizations in SMA type 1 and improvement in SDB symptoms in SMA type 2 and 3, decrease in chest deformity in patients with SMA type 1 and 3 and improvement in sleep quality/architecture and cyclic-alternating patterns in patients with SMA type 1 and 2 (19, 69).

Tracheostomy might offer some advantages in acute scenarios characterized by “mucus plugging” or secretions aspiration resulting in severe desaturation, as it may facilitate swift suctioning and bag-mask ventilation (67, 70). However, risks and benefits should be carefully considered, also from an ethical perspective and discussed with the family, as invasive ventilation would leave the patients with no or minimal communication means in spite of a normal intellectual condition (71).

Common challenges associated with NIV use encompass interface-related pressure sores and midface hypoplasia if NIV is initiated at an early age (49). These concerns are generally manageable through the use of alternative masks with distinct pressure points (16). Aerophagia may happen and it can be mitigated by venting gastric air through a gastrostomy or nasogastric tube. The presence of bulbar palsy can potentially pose complications while using NIV, as it might intensify the accumulation of oral secretions and subsequent aspiration. Potential solutions to counteract this risk include routine suctioning of oral secretions, application of anticholinergic medications for secretion control (e.g., glycopyrronium bromide), employment of nasal masks, and lateral positioning (38).

## 6 Novel SMA treatments and their impact on sleep and respiratory function

The emergence of novel therapeutic approaches for SMA, such as nusinersen and gene therapy, has brought about remarkable prospects for altering the natural progression of the disease and its impact on respiratory function. These therapies aim to address the underlying genetic cause of SMA and potentially slow down disease progression. The effects of novel treatments on respiratory function, sleep and SDB are summarized in Table 1, and discussed in detail in the following paragraphs.

**Table 1:** Effects of currently available SMA treatments on respiratory function, sleep and SBD.

			Effects of therapy on respiratory function								Effects of therapy on sleep and SBD				
			Authors									Authors			
Type of therapy	Commercial name	Molecular mechanism	Finkel et al. (72)	Erdos and Wild (73), Pechmann et al. (74), Finkel et al. (72), Xiao et al. (75), Panagiotou et al. (76), and Heitschmidt et al. (77)	Aragon-Gawinska et al. (78), Lavie et al. (79), Pechmann et al. (80), and Audic et al. (81)	Sansone et al. (82)	Hepkaya et al. (83)	Bertini et al. (84)	Gonski et al. (85) and Shin et al. (86)	Gómez-García de la Banda et al. (87)	Overall observations	Gonski et al. (85)	Chacko et al. (88)	Verrillo et al. (89)	Overall observations
Antisense oligonucleotide	NUSINERSEN	*Targets the SMN2 gene to promote the production of functional SMN protein*	Delays time to invasive ventilation in SMA type 1 patients (Clinical trials)	Respiratory function and time to invasive and non-invasive ventilation did not improve at the same pace as motor skills	The need for respiratory support significantly increased over time (a study on SMA type 1 patients)	Most patients in the cohort (SMA type 1) remained stable (77%). Most patients under 2 years did not require NIV ≥16 hours.	Amelioration of respiratory function in 3/18 SMA type 1 patients, worsening in 5/18 and stability in the remanining. Stability in SMA type 2 (*n*=12) and SMA type 3 (*n*=13) patients.	Treatment in presymptomatic infants at a younger age seems to result in a better respiratory outcome (phase 2 NURTURE study on pre-symptomatic infants)	No significant improvement in the majority of lung function parameters	In children with SMA Type 2, respiratory muscle performance significantly improved after six injections of nusinersen compared with age-matched SMA Type 2 historical controls	Nusinersen has a slight, but positive impact on respiratory function, which differ according to the age of treatment and the severity of the disease, with a greater impact on less compromised patients	No significant improvement in the majority of PSG parameters; in paired analyses: significant improvement in the oxygen nadir; in unpaired analyses: statistical improvement in total apnea-hypopnea index (AHI) and oxygen nadir	Improved SBD (as measured by the AHI)	Short-term effects with an improvement in sleep efficiency and reduction in sleep onset latency; moderate effect in terms of the number of CAP A3 subtypes (showing a slight increase)	Nusinersen leads to a potential improvement in the organization of sleep patterns

			Day et al. (90) and Mendell et al. (91)	Strauss et al. (92)	Pane et al. (93), AlNaimi et al. (94), Mendell et al. (95), D’Silva et al. (96), and Al-Zaidy et al. (97)	Overall observations	Leon-Astudillo et al. (98)	AlNaimi et al. (94)	Overall observations
Gene replacement therapy (GRT)	ZOLGENSMA/onasemnogene abeparvovec	*Introduces a functional copy of the SMN1 gene using an adeno-associated viral vector*	Increases survival and reduces the need for permanent ventilation in SMA type 1 patients (Phase 1 START study and phase 3 STR1VE study)	Initiating treatment in presymptomatic patients has the potential to yield a more favorable prognosis in terms of both motor skill development and respiratory function	Favorable respiratory outcomes in real-world and extension studies (reduced need for respiratory support and reduced hospitalization rate compared to historical cohorts)	GRT treatment seem to have a beneficial impact on respiratory outcome, with an increased number of patients remaining free of ventilation and a general stabilization of those already treated with NIV.	SDB with increased AHI, especially during REM sleep, despite improved neuromotor function.	PSG showed a not statistically significant reduction in both median AHI and REM-AHI. Improvement in total AHI, REM-AHI and respiratory rate (three patients with PSG studies performed both pre- and post-GRT)	Additional studies are needed

			Darras et al. (99)	Baranello et al. (100)	Pascual-Morena et al. (101)	Overall observations	
Splicing modifier	RISDIPLAM	*Increases the systemic levels of functional SMN protein, modifying the splicing of SMN2 pre-messenger RNA*	The percentages of type 1 SMA patients treated who met motor milestones and recorded improvements in motor function were higher than in untreated historical cohorts (Clinical trials); the survival without permanent ventilation of Risdiplam-treated patients was 85%, versus 42% of untreated controls	Increased expression of functional SMN protein in the blood	The efficacy on respiratory function in SMA type 2 and 3 is inconsistent	Improvement in motor function measures compared to historical cohorts in SMA type 1, 2 and 3 patients	No studies available

Nusinersen, an antisense oligonucleotide, targets the *SMN2* gene to promote the production of functional SMN protein. This innovative treatment was able to enhance survival rates, halt the progression of the disease, and even facilitate motor skill advancement. In clinical trials, nusinersen proved able to significantly delay time to invasive ventilation in SMA type 1 patients (72), while respiratory function was not assessed in later-onset SMA (17). Several real-world studies followed, with somewhat conflicting results. In some, it was observed that respiratory function and time to invasive and non-invasive ventilation did not improve at the same pace as motor skills after nusinersen administration, although these outcomes were not monitored carefully (72–77). In some studies on SMA type 1 patients, the need for respiratory support significantly increased over time (78–81). However, treatment at a younger age seems to result in a better respiratory outcome, as shown in the phase 2 NURTURE study on pre-symptomatic infants receiving nusinersen treatment (84). One of the primary endpoint was time to respiratory intervention (invasive and non-invasive ventilation for ≥6 h per day for ≥7 days), and by the age of 25 months no infant was receiving permanent ventilation, in dramatic contrast with natural history studies that show that SMA type 1 infants usually die or require permanent ventilation by the age of 13.5 months. Four (16%) infants needed respiratory intervention during the study period, in the event of an acute, reversible illness. Before the end of the study, two of these infants no longer utilized respiratory intervention, while the other two infants continued to receive NIV for 2 and 10 h per day, respectively. Overall, nusinersen in presymptomatic infants seems to have a huge impact on respiratory function, although no specific respiratory parameter was assessed. In a study by Sansone and colleagues conducted on 118 SMA type 1 (median age 42.8 months), most patients remained stable (82). More than 80% of the children treated before age 2 years survived without the need for NIV ≥16 h. Similarly, a study conducted by Gonski and colleagues in patients with SMA type 1, 2 and 3 showed stability in respiratory function when compared to the natural history of SMA (85). They reported 6 patients with SMA type 2 or 3 (age range 2–17 years) who ceased nocturnal NIV in the two-year period post treatment with nusinersen. Fifteen patients (SMA1 = 3, SMA2 = 8, SMA3 = 3) were already on NIV treatment prior to commencing nusinersen, while 3 patients (SMA1 = 2, SMA2 = 1) were started on NIV after introduction of nusinersen therapy. No significant change in pressure was recorded during the study period. In addition, no significant improvement in the majority of lung function parameters was found (85). Stability of respiratory function and need for ventilation was observed also by Shin and colleagues in a cohort of SMA type 1 patients with permanent ventilation via tracheostomy (86). Conversely, a study conducted on SMA type 2 patients showed an improvement in respiratory function compared to historical controls (87). Hepkaya and colleagues reported of amelioration of respiratory function, with closure of tracheostomy in a patient with SMA type 1 and progressive reduction of time on NIV (<16 h) in two other patients, among a group of 18 SMA type 1 (83). Of those who were initially at room air, three patients underwent tracheostomy and two started to use NIV (>16 h). Seven patients remained stable without oxygen support. Patients with SMA type 2 (*n* = 12) and SMA type 3 (*n* = 13) remained stable without respiratory support during the study period (83). Overall, available studies seem to indicate a slight, but positive impact of nusinersen on respiratory function, which differs according to the age of treatment and the severity of the disease. The benefits appear indeed more evident in SMA type 1 patients treated precociously or in SMA type 2–3 patients. Currently, there exists a lack of extended experience concerning the long-term evolution of respiratory function among patients undergoing nusinersen. The specific respiratory muscles’ response to nusinersen remains unclear and does not follow the motor responses demonstrated. Further studies, including systematic reviews and meta-analyses of current data, are warranted in order to better assess the impact of nusinersen on respiratory function.

Gene replacement therapy (GRT), namely Zolgensma or onasemnogene abeparvovec, aims to introduce a functional copy of the *SMN1* gene using an adeno-associated viral vector. Zolgensma is a one-time intravenous treatment and has shown promising results, particularly in early intervention. Phase 1 START study and phase 3 STR1VE study showed that GRT was able to increase survival and reduce the need for permanent ventilation in SMA type 1 patients (90, 91). In the STR1VE study, 20 out of 22 patients were free of permanent ventilation at the age of 14 months, while in the START study all patients in the therapeutic-dose cohort were free of permanent ventilation at the age of 24 months, in striking contrast with natural history studies. Initiating treatment in presymptomatic patients has demonstrated the potential to yield a more favorable prognosis of both motor development and respiratory function (92). Real-world and extension studies evidence showed favorable respiratory outcomes (93–97). The five-year extension results of the START study show that all 10 patients were alive and free of permanent ventilation 5 years after the treatment; 4 patients in this cohort had required NIV support in the START study and maintained this requirement in the extension study, with no decline in their respiratory status or increased need for baseline respiratory support. AlNaimi and colleagues report the respiratory outcomes of a cohort of 11 patients (9 SMA1, 2 SMA2) treated with GRT. Five SMA type 1 patients required ventilation before GRT, but the four of those who required NIV were successfully weaned off after GRT. The median interval between GRT and complete discontinuation of NIV in these four patients was 10.75 (6–18) months. Unfortunately, one patient underwent tracheostomy and failed weaning. In addition, length of required escalation of respiratory support during acute hospitalizations dropped by 18.56 days/patient/year post-GRT (94). Pane and colleagues report findings from a cohort of 62 SMA1 and 4 presymptomatic patients treated with GRT. As regards the respiratory outcomes, 10 were free of NIV at baseline, and 9 of them remained on spontaneous breathing after 12 months; of the remaining 36 requiring non-invasive ventilation (76%) at baseline, 32 needed NIV for <10 h/day, and 4 for >10 h/day at baseline. Twelve months after treatment, one patient showed a reduction in the duration of daily ventilation to <10 h/day and another one stopped ventilation completely. In the study by D’Silva and colleagues, out of the 21 patients studied, seven required NIV at baseline, but only five continued to require respiratory support after a median of 15 months following onasemnogene abeparvovec. One infant initiated NIV after treatment following recurrent aspiration pneumonia. Overall, these studies show that treatment with onasemnogene abeparvovec seem to have a beneficial impact on respiratory outcome, with an increased number of patients remaining free of ventilation and a general stabilization of those already treated with NIV. Meta-analyses and comparisons with historical cohorts are needed in order to precisely assess the magnitude of this effect.

Risdiplam is another recently approved small molecule for the treatment of SMA, which modifies the splicing of *SMN2* pre-messenger RNA, increasing the systemic levels of functional SMN protein. In clinical trials, the percentages of SMA type 1 patients treated with Risdiplam who met motor milestones and recorded improvements in motor function were higher than in untreated historical cohorts (99). In addition, the survival without permanent ventilation of Risdiplam-treated patients was 85%, versus 42% of untreated controls (99). Patients also showed an increased expression of functional SMN protein in the blood (100). A recent systematic review and meta-analysis assessed the efficacy and safety of Risdiplam in SMA patients in 11 published studies, showing that, while these studies overall show an improvement in motor function measures compared to historical cohorts in SMA type 1, 2 and 3 patients, the efficacy on respiratory function in SMA type 2 and 3 patients was inconsistent (101).

As regards the impact of novel therapies on sleep and SDB, available data are scarce (Table 1). Gonski and colleagues assessed sleep outcomes in children with SMA treated with nusinersen (85). No significant improvement in the majority of PSG parameters (such as mean SpO2 or SpO2below 90%) were found. However, analyzing polysomnographic studies, the authors found a significant improvement in the oxygen nadir in paired analyses (87.9% pre-nusinersen to 92.3% post-nusinersen; *p* = 0.01), while unpaired analyses showed a statistical improvement in total AHI (reduced from 13.96 to 2.9 events/h, *p* = 0.03) and oxygen nadir (increased from 88.09 to 91.45%, *p* = 0.01) when comparing pre and post nusinersen diagnostic sleep studies’ results (85). As nusinersen results in a better motor function, the authors speculate that the improvement in the oxygen nadir could result from an increase in the tone of the upper airway dilator skeletal muscle, improved coughing and better airway clearance, resulting in better ventilation and oxygenation in sleep (85). These findings are similar to those observed by Chacko and colleagues, which found that treatment with nusinersen improved SDB, as measured by the AHI (88). Conversely, Verrillo and colleagues observed short-term effects of nusinersen on sleep with an improvement in sleep efficiency and reduction in sleep onset latency; regarding sleep microstructure, a moderate effect was observed in terms of the number of CAP A3 subtypes, showing a slight increase (89). This discovery suggests the potential for an overall improvement in the organization of sleep patterns linked to the treatment. Nevertheless, all these results require validation through extensive studies involving patients who commenced treatment at an earlier age and over an extended treatment duration. As regards GRT, AlNaimi and colleagues assessed the presence of SDB in a cohort of 11 patients (9 SMA1, 2 SMA2) treated with Zolgensma (94). Before GRT, PSG (performed in 4 patients with a median age of 6 months), showed an increase in AHI (median 16.7 events/h) and REM-AHI (median 28.3 events/h), with mainly obstructive events. After GRT, PSG (performed in 8 patients at a median age of 22 months) displayed a reduction in both median AHI and REM-AHI (respectively, 4.6 events/h and 15.9 events/h). However, the reduction was not statistically significant. Only three patients had PSG studies performed both pre- and post-GRT, and improvement in total AHI, REM-AHI and respiratory rate was observed. A study by Leon-Astudillo and colleagues assessed PSG finding in 8 children with SMA who received GRT with onasemnogene abeparvovec (98). Two children of the cohort also received nusinersen and risdiplam before GRT, respectively. All subjects had SDB with increased AHI, especially during REM sleep, despite improved neuromotor function. A limitation of this study is that PSG was only performed after starting the treatment, so comparisons between pre- and post-treatment variables cannot be made. In addition, time from treatment to PSG varies widely among the cohort, spanning between 1 and 13.3 months, making it difficult to draw precise conclusions regarding the impact of treatment on SDB in this cohort. Overall, additional studies with bigger sample sizes and pre- and post-treatment studies are needed in order to observe a potential statistically significant impact of GRT on these parameters. Unfortunately, no studies are available regarding the impact of risdiplam on sleep function and SDB in SMA patients.

While challenges and questions remain, such as the optimal timing of intervention and the long-term durability of therapeutic effects, these therapies represent a significant stride toward mitigating the burden of SMA and enhancing the overall well-being of affected individuals. These considerations notwithstanding, the impact of these treatments on respiratory function and on the need for ventilatory support, remain subjects of ongoing research.

## 7 Conclusion

In conclusion, the management of SMA, a complex genetic disorder affecting motor neurons, poses significant challenges and necessitates a comprehensive, multidisciplinary approach. Sleep-related breathing disorders and respiratory complications are common in SMA, particularly in SMA type 1 patients, where respiratory impairment is most severe. The delicate balance between muscle weakness, respiratory load, and ventilatory support underscores the critical importance of vigilant monitoring and timely interventions to preserve pulmonary function and enhance the overall quality of life for SMA patients.

Sleep monitoring in SMA presents unique challenges due to the need for specialized equipment and expertise. Polysomnography remains the gold standard, but its practicality and accessibility are limited, especially for disabled patients. Alternative methods, such as respiratory polygraphy, offer simpler and effective means of studying sleep in SMA patients, although diagnostic criteria for hypoventilation require careful consideration.

NIV plays a pivotal role in managing respiratory complications in SMA, providing respiratory support while preserving speech abilities and quality of life. However, challenges like interface-related pressure sores and midface hypoplasia must be addressed. The timing of NIV initiation varies based on clinical indications, but early intervention may help alleviate dyspnea and prepare patients for potential acute deterioration.

Recent advancements in SMA treatment, including nusinersen and GRT (Zolgensma), hold promise in altering the disease’s natural progression. While these therapies primarily target motor function, their impact on respiratory function and sleep quality is a subject of ongoing research. Early evidence suggests potential benefits, but more extensive studies are needed to validate these findings.

In summary, SMA management necessitates a holistic approach that includes vigilant sleep monitoring, timely intervention with NIV, and the integration of emerging therapies to enhance both motor and respiratory function. The future holds promise for improving the quality of life and outcomes for individuals with SMA, but continued research and clinical experience are essential to fully understand the long-term effects of these treatments on sleep and respiratory function. Indeed, despite the importance of respiratory support in SMA, knowledge on sleep disorders in this population is limited. The evolving landscape of SMA therapeutics demonstrates the potential for substantial improvements in respiratory outcomes, allowing individuals with SMA to lead more fulfilling lives by mitigating the burdens of compromised respiratory function.

With a diverse spectrum of respiratory involvement across different SMA types, close monitoring, early intervention, and tailored ventilatory support are essential to ensure optimal outcomes. As research continues to uncover the underlying mechanisms of the disease and novel therapies emerge, the management of respiratory complications in SMA is poised to evolve, offering hope for improved quality of life for affected individuals.

## Author contributions

EA: Writing – review & editing, Writing – original draft, Supervision, Project administration, Investigation. EM: Writing – review & editing, Writing – original draft, Investigation, Conceptualization. MR: Writing – original draft. BM: Writing – review & editing, Writing – original draft, Investigation. FP: Writing – review & editing, Supervision. GC: Writing – review & editing, Supervision, Resources, Funding acquisition. SC: Writing – review & editing, Supervision, Resources, Project administration, Funding acquisition, Conceptualization.
